# Supplementary material for: Nucleotide resolution mapping of influenza A virus nucleoprotein-RNA interactions reveals RNA features required for replication
Source: Nat Commun. 2018 Jan 31;9:465. doi: 10.1038/s41467-018-02886-w (PMC5792457; doi:10.1038/s41467-018-02886-w)
Supplement: Supplementary file 1 — Supplementary Information [file 41467_2018_2886_MOESM1_ESM.pdf]

Supplementary Information

Nucleotide resolution mapping of Influenza A virus nucleoprotein-RNA interactions reveals RNA features required for replication

Williams G.D. et al.

Supplementary Figure 1.

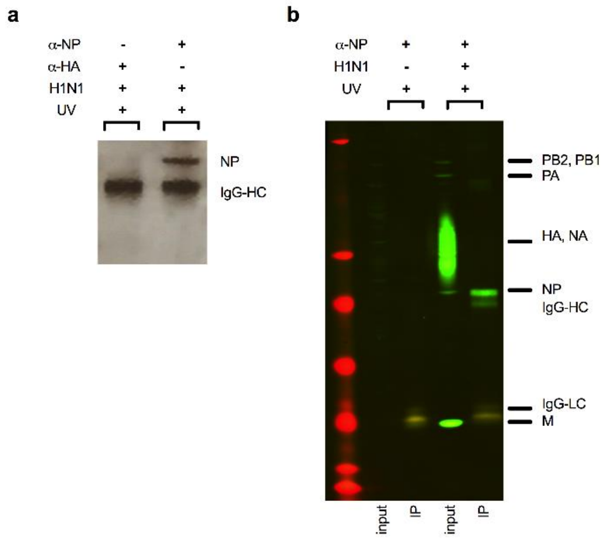

NP immunoprecipitation protocol results in pure NP elution. a, Silver stain of immunoprecipitation (IP) eluate for IAV infected cells with either anti-HA or anti-NP antibodies. The upper band represents NP and lower band represents IgG heavy chain. Gel image is representative of two independently performed experiments **b,** IP of NP from purified virus particles removes all other viral proteins. Western blot using polyclonal goat sera against H1N1 virus to detect all viral proteins in input and NP-immunoprecipitated eluate. Lane 1 is a marker, lanes 2 and 3 are control immunoprecipitation beads in which no virus lysate was added. Lanes 4 and 5 are input or IP eluate from virus lysate immunoprecipitated with anti-NP antibody HB65. The membrane was probed with goat-anti-IAV USSR (H1N1) (US Biological, I7650-78B) and IRDye 800CW donkey-anti-goat (Licor). Gel image is representative of two independently performed experiments. Original western-blot and silver stain are shown in Supplementary Figure 7.

Supplementary Figure 2.

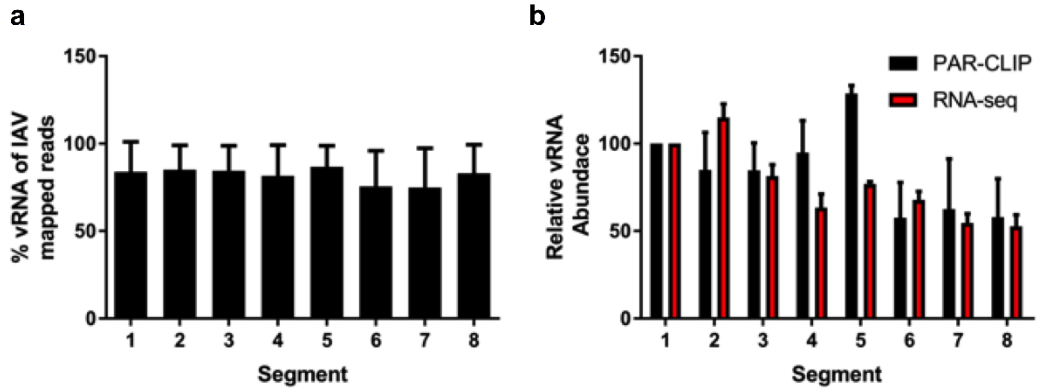

Percentage of mapped reads derived from negative-sense viral RNA (vRNA) for four PAR-CLIP experiments (mean + S.E.M., n = 4). **a,** The total number of base calls for reads mapping to the negative and positive strand were calculated and the number of base calls from negative strand (vRNA) is plotted as a percentage of total base calls. PAR-CLIP and RNA-seq sequence abundance per gene segment (vRNA only) of IAV. Calculated as the total number of base calls per segment and normalized to segment 1 (mean + S.E.M., n = 4 each). **b,** Total base calls for each vRNA was calculated and compared between PAR-CLIP and RNA-seq using segment 1 as an internal normalization standard.

Supplementary Figure 3.

a

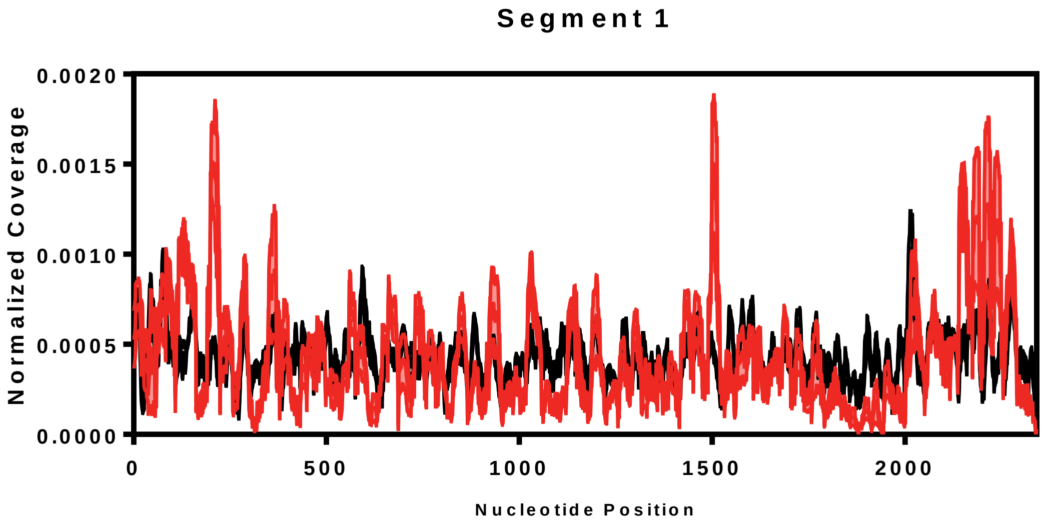

b

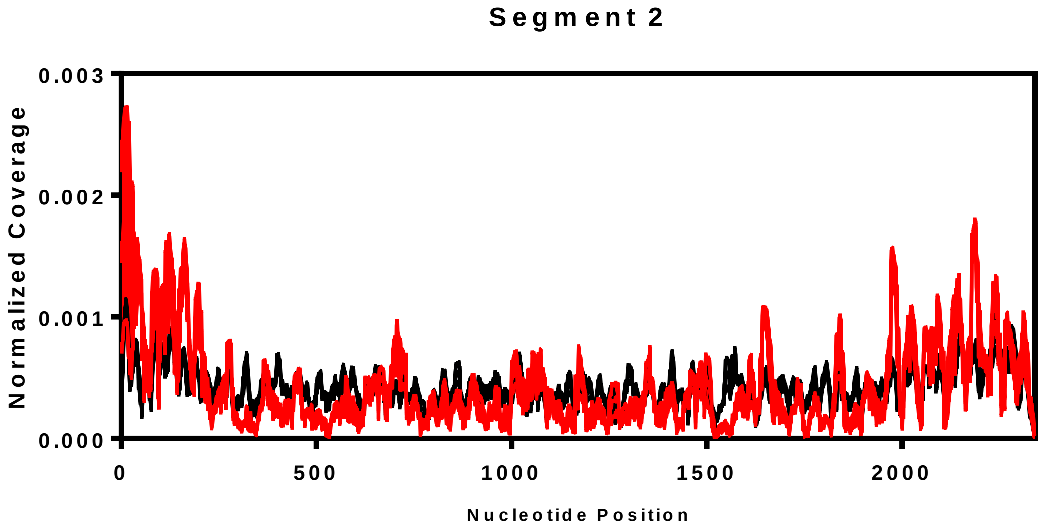

c

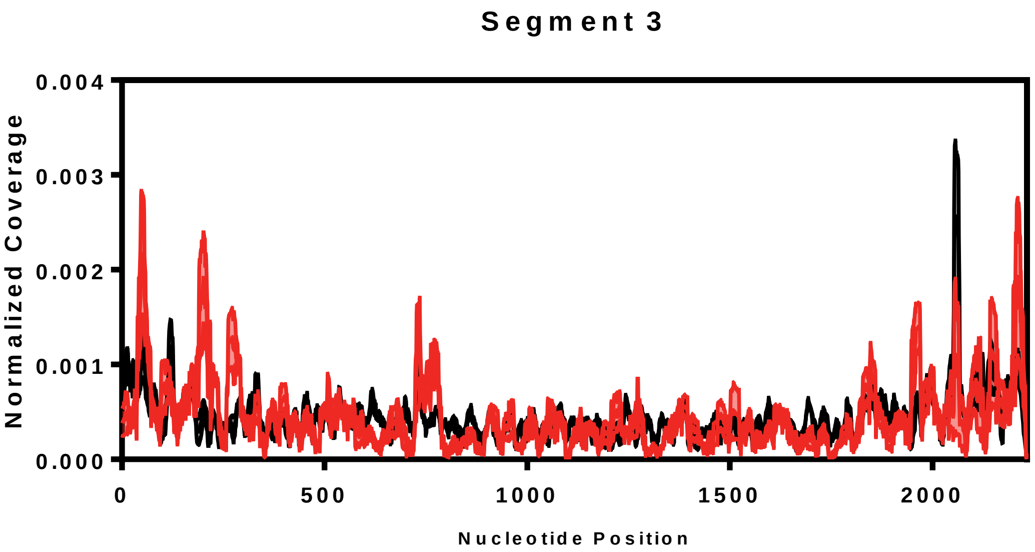
Segment 3
0.004
0.003
0.002
0.001
0.000
Normalized Coverage
0
500
1000
1500
2000
Nucleotide Position

d

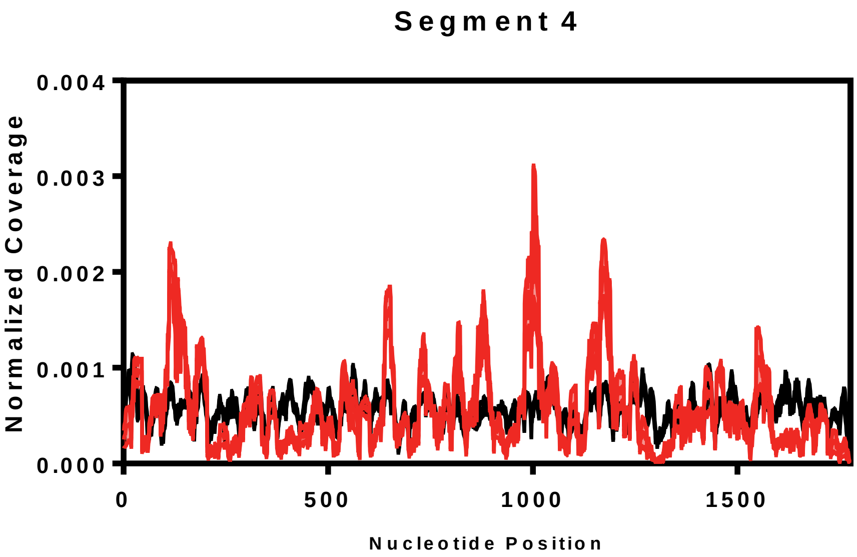
Segment 4
0.004
0.003
0.002
0.001
0.000
Normalized Coverage
0
500
1000
1500
Nucleotide Position

e

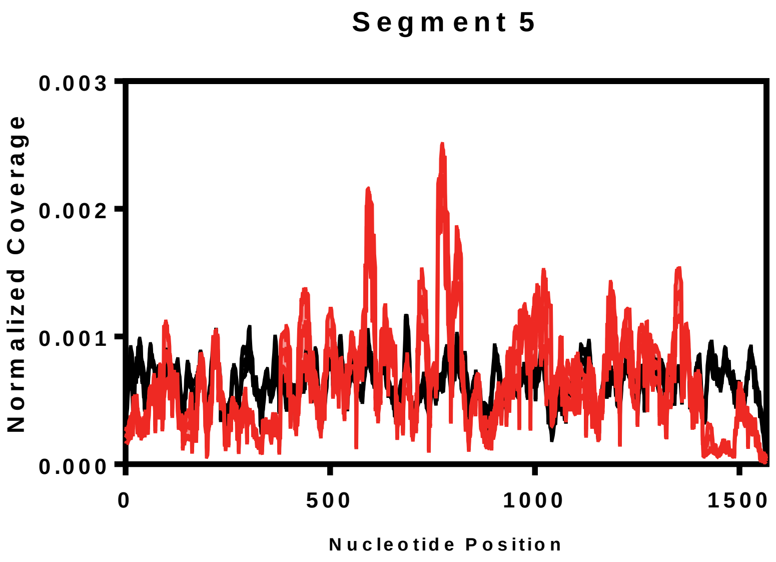
Segment 5
Normalized Coverage
0.003
0.002
0.001
0.000
0
500
1000
1500
Nucleotide Position

f

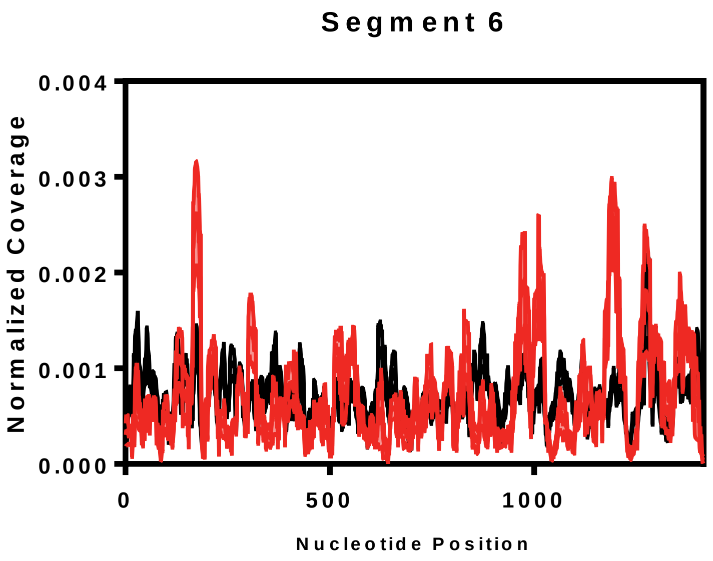
Segment 6
Normalized Coverage
0.004
0.003
0.002
0.001
0.000
0
500
1000
Nucleotide Position

g

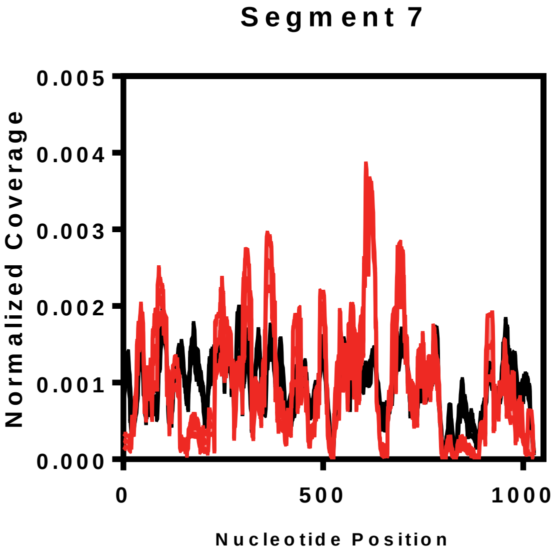

h

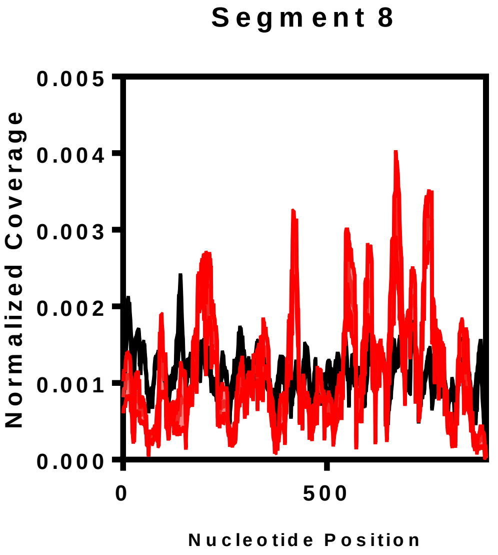

a-h, Normalized coverage for complete genome segments for PAR-CLIP (red) and RNA-seq (black). Lines represent mean +/- standard deviation S.E.M. from four experiments each.

Supplementary Figure 4.

a

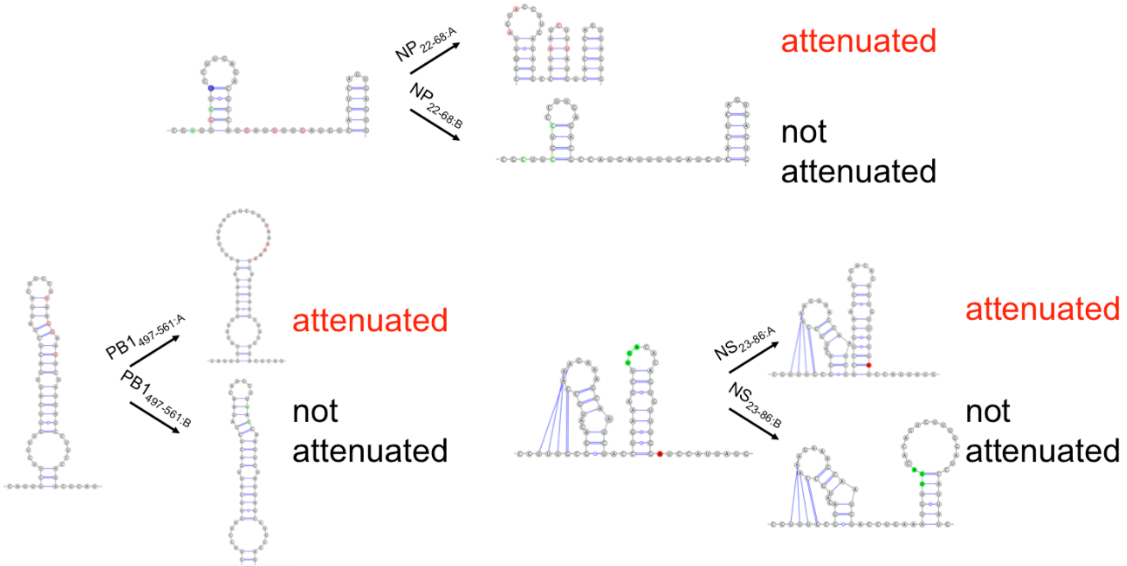

b

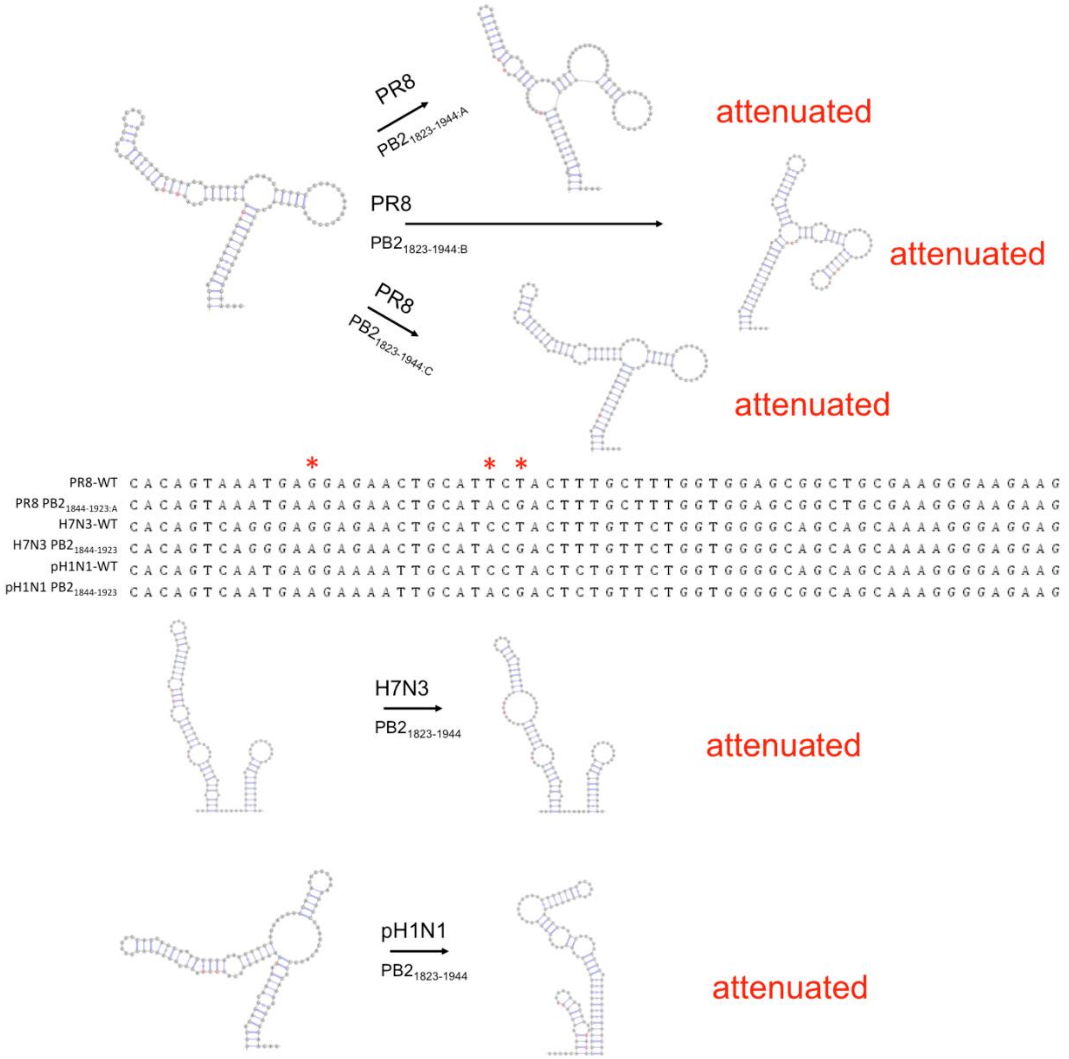
PR8
PB2 1823-1944:A
attenuated
PR8
PB2 1823-1944:B
attenuated
PR8
PB2 1823-1944:C
attenuated
*
PR8-WT CACAGTAAATGAGGAGAACTGCATTCTACTTTGCTTTGGTGGAGCGGCTGCGAAGGGAAGAAG
PR8 PB2 1844-1923:A CACAGTAAATGAAGAGAACTGCATACGACTTTGCTTTGGTGGAGCGGCTGCGAAGGGAAGAAG
H7N3-WT CACAGTCAGGGAGGAGAACTGCATCCTACTTTGTTCTGGTGGGGCAGCAGCAAAAGGGAGGAG
H7N3 PB2 1844-1923 CACAGTCAGGGAAGAGAACTGCATACGACTTTGTTCTGGTGGGGCAGCAGCAAAAGGGAGGAG
pH1N1-WT CACAGTCAATGAGGAAAATTGCATCCTACTCTGTTCTGGTGGGGCAGCAGCAAAGGGGAGAAG
pH1N1 PB2 1844-1923 CACAGTCAATGAAGAAAATTGCATACGACTCTGTTCTGGTGGGGCGGCAGCAAAGGGGAGAAG
H7N3
PB2 1823-1944
attenuated
pH1N1
PB2 1823-1944
attenuated

c

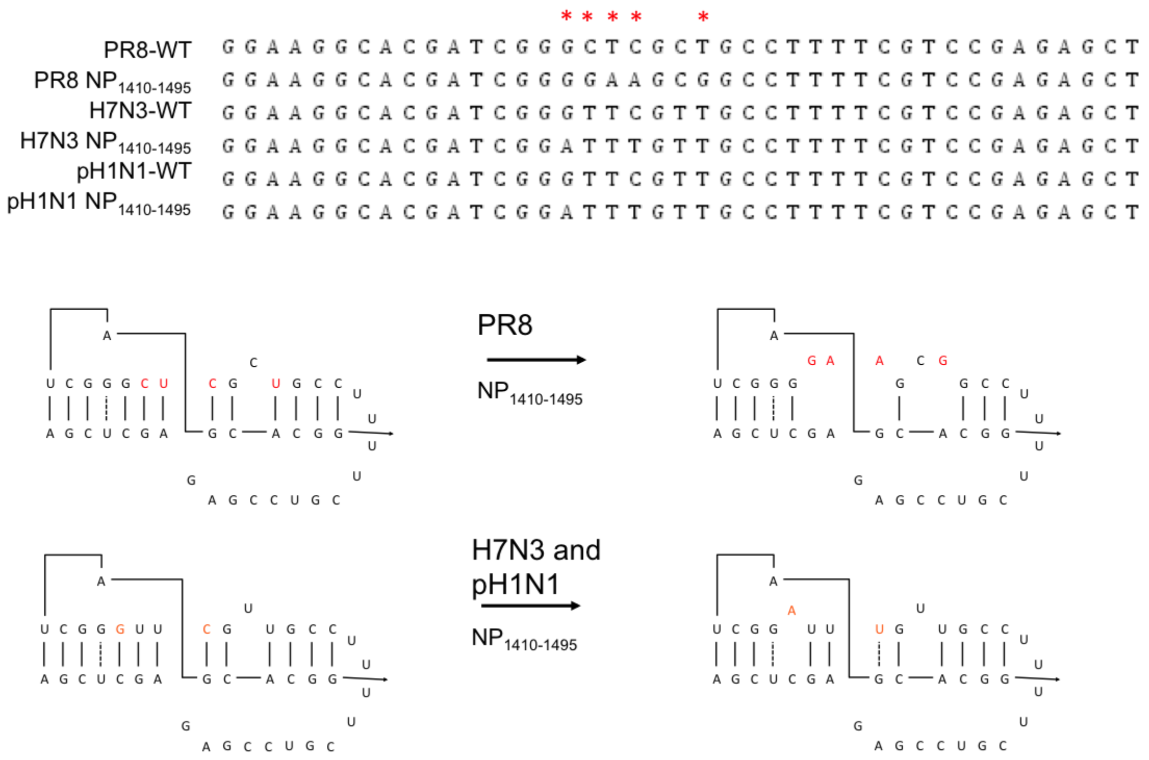

d

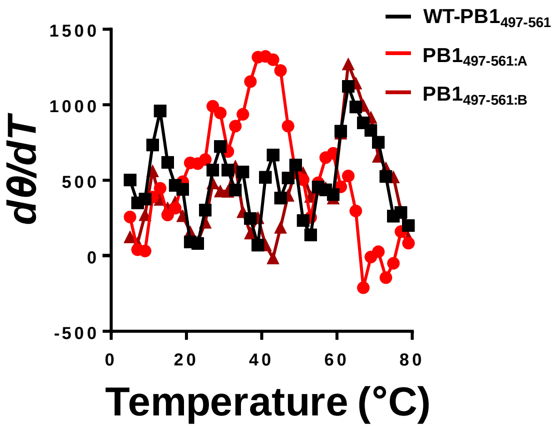

Predicted RNA structures of WT-PR8, WT-H7N3, and WT-pH1N1 IAV and the indicated mutant viruses. The nucleotides where we introduced substitutions are highlighted with a red star in alignments for a portion of the $PB2_{1823\text{-}1944}$ and $NP_{1410\text{-}1495}$ regions. In all examples, the WT structures are to the left of the arrow and mutants to the right. All structures represent the vRNA minimum free energy structure prediction using base settings from the RNAfold server, visualized using VARNA. **a,** Predicted secondary structures in the vRNA regions of $NP_{22\text{-}68}$, $PB1_{497\text{-}561}$, and $NS_{23\text{-}86}$. The attenuated mutant A viruses contain RNA structure destabilizing synonymous mutations, whereas non-

attenuated mutant B contain control synonymous mutations. **b,** Predicted secondary structures in the vRNA region of $PB2_{1823-1944}$ of PR8, H7N3 and pH1N1 IAV. **c,** Alteration of a previously identified pseudoknot within the $NP_{1410-1495}$ vRNA region of WT-PR8 and WT-H7N3 and WT-pH1N1 IAV. **d**, Thermal denaturation of $PB1_{497-561,}$ $PB1_{497-561:A,}$ and $PB1_{497-561:B}$ RNA as measured by CD at 210 nm. RNA was heated from 5°C to 95°C at a rate of 1°C/min and readings were collected every 2°C to monitor unfolding. Data is presented as the change in molar ellipticity as a function of temperature (dθ/dT). One representative experiment of two is shown.

Supplementary Figure 5.

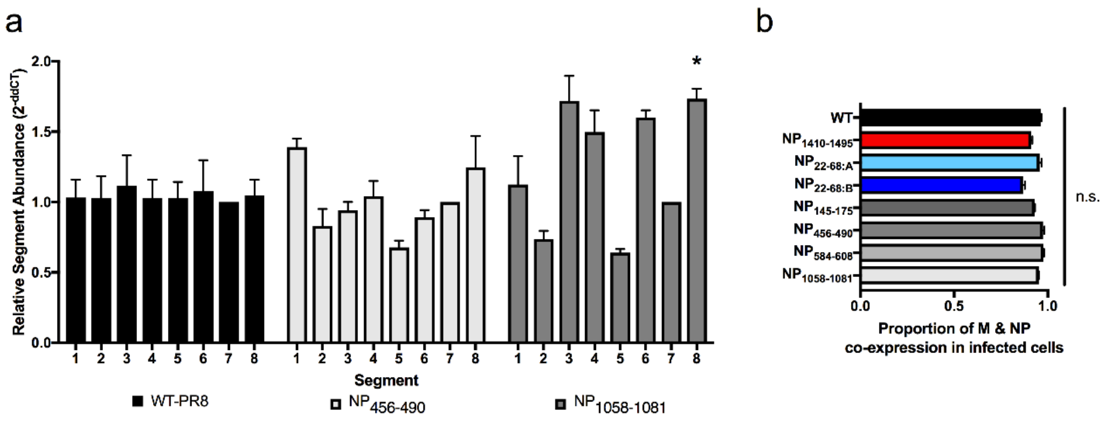

a, Packaging qPCR of additional mutant viruses, as in Figure 3f (*, $P < 0.05$). The relative abundance of each genome segment in concentrated virus particles was assessed and normalized to that of segment 7 and WT-PR8 virus by the 2^{-ddCT} method as stated in the materials and methods. Results are the average of three experiments. * is $P<0.05$ by one-way ANOVA with multiple comparisons correction (Kruskal-Wallis test). **b,** co-expression of M and NP proteins in MDCK cells infected at high MOI (5) for 16 hours. High MOI infection of MDCK cells overcomes co-expression defects observed at low MOI (Figure 3e). Proportion of infected cells co-expressing of NP and M was determined by calculating the number of NP+M+ double positive cells and dividing by the total number of cells expressing NP or M when assessed by flow cytometry. Results are derived from two independent experiments performed in duplicate. n.s. is not significant by one-way ANOVA with multiple comparisons correction (Kruskal-Wallis test).

Supplementary Figure 6.

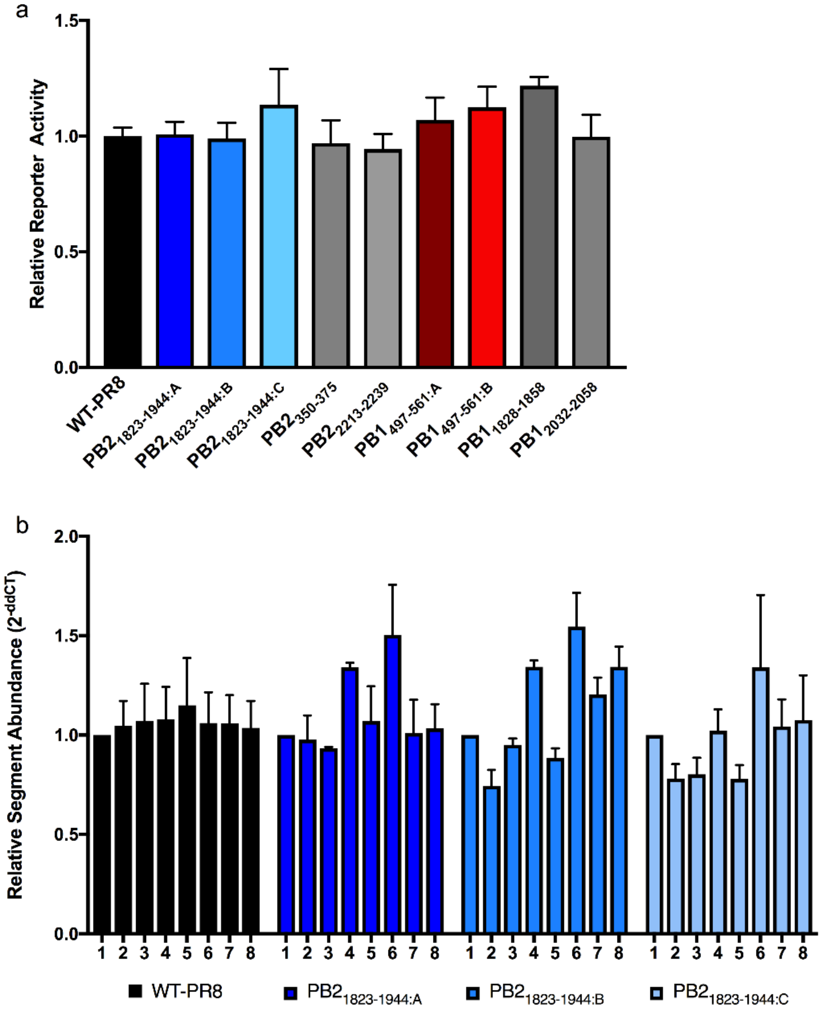

a, Dual-luciferase reporter assay to assess viral transcription and genome replication, no significant differences observed by one-way ANOVA with multiple comparisons correction (Kruskal-Wallis test). The results are the average + S.E.M. of three experiments performed in duplicate. **b,** Relative abundance of genome segments in purified WT or mutant viruses. all segments were compared to segment 1 (PB2) vRNA and normalized to the average of WT-PR8 values using the 2^{-ddCt} method[22]. Results are the average of 3-6 independent virus preparations + S.E.M..

Supplementary Figure 7.

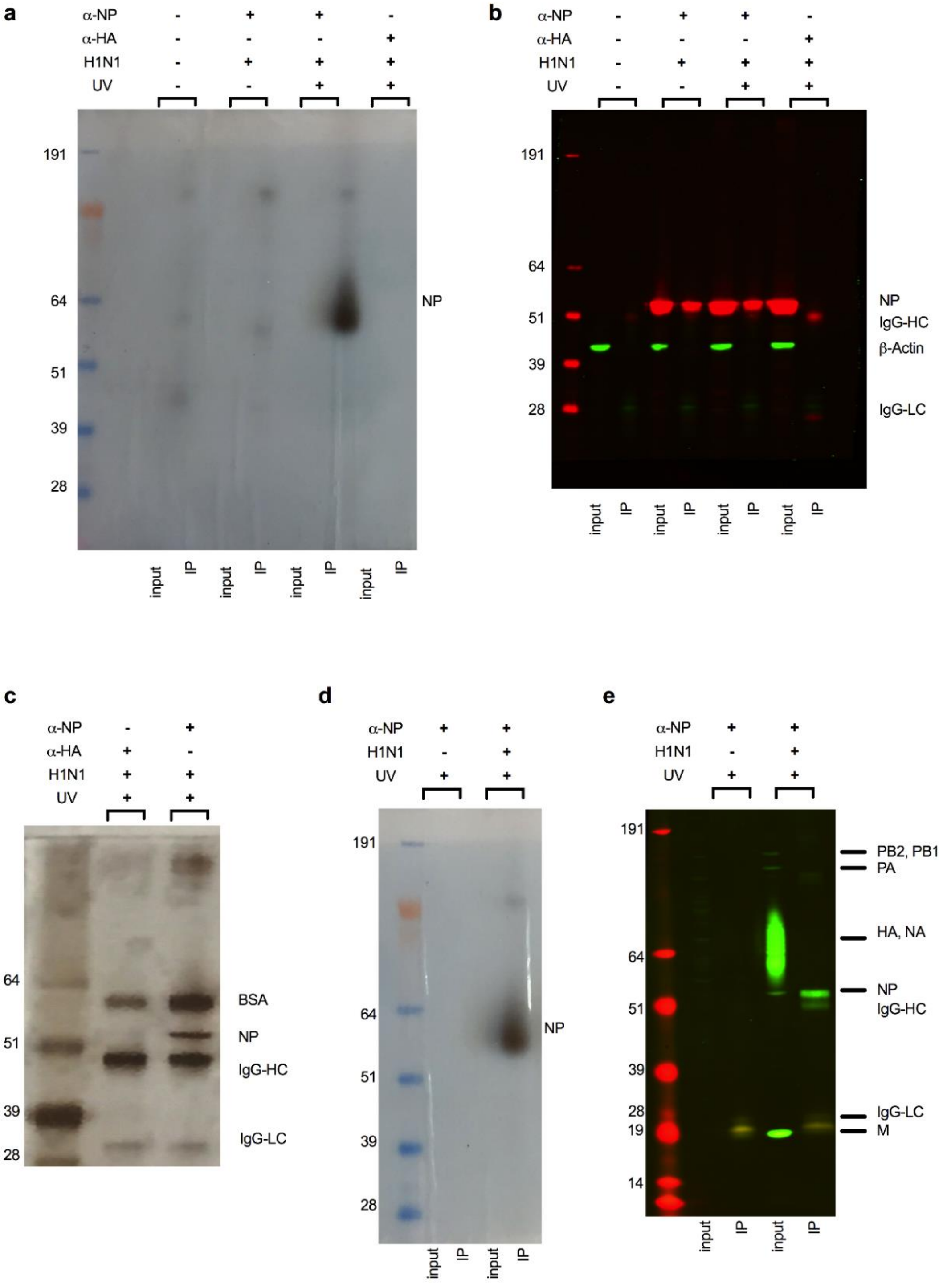

Original western-blots, silver stain and autoradiographs shown in Figure 1 and Supplementary Figure 1.

Supplementary Table 1.

Segment 1				
**Low (nt)**	**FDR min**	**Fold-change**	**Length**	**MFE**
307-333*	0.01	>3	>18	0.0
406-431	0.05	>3	>18	-5.3
1089-1121	0.05	>3	>18	-2.2
1823-1944*	0.01	>3	>18	-33.0
2321-2341	0.05	>3	>18	-1.7
**High (nt)**	**FDR min**	**Fold-change**	**Length**	**MFE**
117-150	0.01	<3	>18	-0.5
2141-2159	0.05	>3	18	-1.2

Segment 2				
**Low (nt)**	**FDR min**	**Fold-change**	**Length**	**MFE**
297-419*	0.01	>3	>18	-30.8
465-482	0.01	<3	18	0.0
497-561*	0.01	>3	>18	-12.9
930-955*	0.01	>3	>18	-2.2
1294-1314	0.05	>3	>18	-0.4
1423-1440*	0.01	>3	18	0.0
1521-1576	0.05	>3	>18	-10.8
1746-1773*	0.01	>3	>18	-0.3
1790-1817*	0.01	>3	>18	-5.2
**High (nt)**	**FDR min**	**Fold-change**	**Length**	**MFE**
39-56*	0.01	>3	18	0.0
2178-2195	0.01	<3	18	-1.2

Segment 3				
**Low (nt)**	**FDR min**	**Fold-change**	**Length**	**MFE**
619-640	0.05	>3	>18	0.0
792-817	0.05	>3	>18	0.0
1098-1113	0.01	>3	16	0.0

Segment 4				
**Low (nt)**	**FDR min**	**Fold-change**	**Length**	**MFE**
208-240*	0.01	>3	>18	-2.1
252-282	0.05	>3	>18	-8.5
376-430*	0.01	>3	>18	-9.0
441-464	0.05	>3	>18	-4.9
491-528	0.05	>3	>18	-1.4
602-628	0.05	>3	>18	-5.0
1256-1340*	0.01	>3	>18	-2.1
1585-1661*	0.01	>3	>18	-14.5
1719-1770*	0.01	>3	>18	-6.0
**High (nt)**	**FDR min**	**Fold-change**	**Length**	**MFE**
98-151*	0.01	>3	>18	-12.3
980-1009	0.05	>3	>18	-1.7
1446-1463	0.01	<3	18	-2.0

Segment 5				
**Low (nt)**	**FDR min**	**Fold-change**	**Length**	**MFE**
22-68*	0.01	>3	>18	-8.7
261-329*	0.01	>3	>18	-12.4
337-378*	0.01	>3	>18	-10.9
1410-1495*	0.01	>3	>18	-19.8
1514-1560*	0.01	>3	>18	-3.3
**High (nt)**	**FDR min**	**Fold-change**	**Length**	**MFE**
634-661	0.01	<2	>18	-1.6
766-787*	0.01	>3	>18	0.0

Segment 6				
**Low (nt)**	**FDR min**	**Fold-change**	**Length**	**MFE**
249-269*	0.01	>3	>18	-6.3
849-866	0.05	>3	18	0.0
1038-1057*	0.01	>3	>18	0.0
1080-1197	0.05	>3	18	0.0
**High (nt)**	**FDR min**	**Fold-change**	**Length**	**MFE**
1181-1199	0.05	>3	>18	0.0

Segment 7				
**Low (nt)**	**FDR min**	**Fold-change**	**Length**	**MFE**
1-16	0.01	>5	16	-1.1
142-201*	0.01	>3	>18	-16.2
640-660*	0.01	>3	>18	-2.5
839-866	0.05	>3	>18	0.0
**High (nt)**	**FDR min**	**Fold-change**	**Length**	**MFE**
601-632*	0.01	>3	>18	-3.2

Segment 8				
**Low (nt)**	**FDR min**	**Fold-change**	**Length**	**MFE**
23-86*	0.01	>3	>18	-10.0
113-139	0.05	>3	>18	-4.8
867-885*	0.01	>3	>18	-0.4
**High (nt)**	**FDR min**	**Fold-change**	**Length**	**MFE**
413-430	0.05	>3	>18	-1.6

Regions of vRNA greater than or equal to 16 nucleotides long and meeting at least two of the three criteria described in the methods. NP-low indicates the region was significantly lower in PAR-CLIP than RNA-seq and NP-high indicates the region was significantly higher in PAR-CLIP than RNA-seq. FDR min is the minimum False

Discovery Rate for each region. Fold-change is absolute fold-difference between PAR-CLIP and RNA-seq data. MFE = Predicted Minimum Free Energy (kcal/mol). * Region meeting all three criteria for a high or low-NP binding region.

Supplementary Table 2.

DNA primers and RNA oligomers used in this study.

Primer	Sequence
**PR8:NP 1410-1495 Forward**	AATACACCTGCTGCTCGAAAAGGCCGCTTCCCCGATCG
**PR8:NP 1410-1495 Reverse**	TTAACACCTGCTGCTTTCGTCCGAGAGCTC
**PR8:NP 22-68:A Forward**	AATTCACCTGCGCTTAGACGCCATAATCTTTATGTCACTCAG
**PR8:NP 22-68:A Reverse**	TTAACACCTGCGCTTGTCTCAAGGTACTAAACGATCTTACGAACAG
**PR8:NP 22-68:B Forward**	TTAACACCTGCGCTTAGACGCCATGATTTTGATGTCACTCAG
**PR8:NP 22-68:B Reverse**	TTAACACCTGCGCTTGTCTCAAGGGACGAAGCGATCTTACGAACAG
**PR8:NP 145-175 Forward**	AATTCACCTGCGCTTATCGTCCAATTCCACCAATC
**PR8:NP 145-175 Reverse**	TTAACACCTGCGCTTCGATTCTATATACAAATGTGC
**PR8:NP 456-490 Forward**	TACACCTGCCTGCCCTCAACGATGCAACTTATCAGAGGACAAGAGCTC
**PR8:NP 456-490 Reverse**	TACACCTGCCTGCGAGGTTACTATGCCAGATCATCATGTGAGTCAGAC
**PR8:NP 584-608 Forward**	TACACCTGCCTGCGTAAAGGGAGTTGGAACAATGGTGATGGAATTGGTC
**PR8:NP 584-608 Reverse**	TACACCTGCCTGCTTACAGCCGCACCTGCGGCTCCAGACCTCCTAGG
**PR8:NP 1058-1081 Forward**	TACACCTGCCTGCCTACGTGTGCTGTCATTCATCAAAGGGACGAAGGTGC
**PR8:NP 1058-1081 Reverse**	TACACCTGCCTGCGTAGATCTTCAAATGCGGCAGAATGGCATGCC
**PR8:PB2 1823-1944:A Forward**	TATACACCTGCGCTTGCAAAGTCGTATGCAGTTCTCTTCATTTACTGTGAATGTG
**PR8:PB2 1823-1944:A Reverse**	AATTCACCTGCGCTTTTGCTTTGGTGGAGCGGC
**PR8:PB2 1823-1944:B Forward**	TTAACACCTGCGCTTTGAGGGACGTGCTTGGGACATTTGATACCGCGCAAATAATAAAAC
**PR8:PB2 1823-1944:B Reverse**	AATTCACCTGCGCTTCTCATTTGTTGGAACAGAGTTCTTAC
**PR8:PB2 1823-1944:C Forward**	TATACACCTGCGCTTGCAAGCAAAGTAGAATGCAGTTCTCCTCATTTATTGTGAAGTG
**PR8:PB2 1823-1944:C Reverse**	AATTCACCTGCGCTTTTGCTTTGGTGGAGCGGC
**PR8:PB1 497-561:A Forward**	AATTCACCTGCAATTGCTCATAGATTTTTTAAAGGATGTAATG
**PR8:PB1 497-561:A Reverse**	TTAACACCTGCTGCTGAGCCTTCCAGACTCATTGG
**PR8:PB1 497-561:B Forward**	TATACACCTGCTGATCTTCCTAAAGGATGTTATGGAGTCAATGAACAAGGAAGAAATG
**PR8:PB1 497-561:B Reverse**	TATACACCTGCTGATGAAGTCTATGAGCCTTCCAGACTCATTGGC
**PR8:NS 23-86:A mutant**	TATACACCTGCCTGCGACAAAAACATAATGGACCCAAACACTG
**PR8:NS 23-86:A mutant**	TATACACCTGCCTGCTGTCACCTGCTTTTGCTCCC
**PR8:NS 23-86:B mutant**	TATACACCTGCCTGCGTAGATTGCTTTCTGTGGCATGTCCGCAAACGAG
**PR8:NS 23-86:B mutant**	TATACACCTGCCTGCTACCTGAAAGCTTGACACAGTGTTTGG
**PR8:PB2 350-375 Forward**	TATACACCTGCTGATGTTCATTATCCAAAAATCTACAAAACTTATTTTGAAAGAG
**PR8:PB2 350-375 Reverse**	TATACACCTGCTGATGAACTGTATTTGTTATTGGTCCATTCCTATTCCACC
**PR8:PB2 2213-2250 Forward**	TATACACCTGCTGATGGAGACGTGGTGTTGGTAATGAAACGGAAACGGGACTC
**PR8:PB2 2213-2250 Reverse**	TATACACCTGCTGATCTCCTTGCCCAATTAGCACATTAGCCTTCTCTCC
**PR8:PB1 2032-2058 Forward**	TATACACCTGCTGATAGAAATCGATCCATCTTGAATACAAGTCAAAGAGGCAAAGAGG
**PR8:PB1 2032-2058 Reverse**	TATACACCTGCTGATTTCTTTTGGGGATCCAGGAGTGTGTTG
**PR8:PB1 1828-1858 Forward**	TATACACCTGCTGATAGAAATCTCCACATTCCTGAAGTCTGCCTAAAATGG
**PR8:PB1 1828-1858 Reverse**	TATACACCTGCTGATTTCTAATGTTGTATAAATTTGGGCCTCC
**H7N3:PB2 1823-1944 Forward**	TATACACCTGCTATAGCAGTTCTCTTCCCTGACTGTGAATG
**H7N3:PB2 1823-1944 Reverse**	TATACACCTGCTATACTGCATACGACTTTGTTCTGGTG
**pH1N1:PB2 1823-1944 Forward**	TATACACCTGCTATAGCAATTTTCTTCATTGACTGTGAATG
**pH1N1:PB2 1823-1944 Reverse**	TATACACCTGCTATATTGCATACGACTCTGTTCTGGTG
**H7N3:NP 1410-1495 Forward**	TATACACCTGCTATACAACAAATCCGATCGTGCCTTC
**H7N3:NP 1410-1495 Reverse**	TATACACCTGCTATAGTTGCCTTTTCGTCCGAGAGCTCG
**pH1N1:NP 1410-1495 Forward**	TATACACCTGCTATACAACAAATCCGATCGTGCCTTC
**pH1N1:NP 1410-1495 Reverse**	TATACACCTGCTATAGTTGCCTTTTCGTCCGAGAGCTCG
**RNA oligomers**	
**PR8:PB1 497-561**	TTCTTCTTTGTTCATTGACTCCATTACATCCTTAAGGAAGTCTATGAGCCTTCCAGACT
**PB1 497-561:A mutant**	TTCTTCTTTGTTCATTGACTCCATTACATCCTTTAAAAATCTATGAGCCTTCCAGACT
**PB1 497-561:B mutant**	TTCTTCCTTGTTCATTGACTCCATAACATCCTTTAGGAAGTCTATGAGCCTTCCAGACT
