# Supplementary material for: Nucleotide resolution mapping of influenza A virus nucleoprotein-RNA interactions reveals RNA features required for replication
Source: Nat Commun. 2018 Jan 31;9:465. doi: 10.1038/s41467-018-02886-w (PMC5792457; doi:10.1038/s41467-018-02886-w)
Supplement: Supplementary file 3 — Description of Additional Supplementary Files [file 41467_2018_2886_MOESM3_ESM.pdf]

Description of Additional Supplementary Files

File Name: **Supplementary Data 1**

Description: **Sequence and predicted RNA structure information of low-NP binding and NP-bound regions analyzed in this study.** Page 1. IAV-PR8 segment 5 vRNA regions and mutations made in viruses utilized in Figures 2 and 3. Page 2 contains IAV-PR8 mutations in segments 1, 2, and 8 made in viruses assessed in Figure 4. Page 3. IAV-H7N3 mutations in segments 1 and 5 introduced to viruses utilized in Figure 5. Page 4. IAV-pH1N1 mutations in segments 1 and 5 introduced to viruses utilized in Figure 5. Nucleotides highlighted in red are synonymous substitutions in the indicated mutant viruses. MFE = Predicted Minimum Free Energy (kcal/mol); Predicted EFE = Ensemble Free Energy (kcal/mol); and Ensemble Diversity calculated using RNAfold. Pseudoknot potential was predicted using vsfold5.
